# Identification and investigation of a novel NADP^+^-dependent secoisolariciresinol dehydrogenase from *Isatis indigotica*

**DOI:** 10.3389/fpls.2022.1035121

**Published:** 2022-11-02

**Authors:** Xiaoyi Shi, Jiaran Geng, Jingxian Feng, Yingbo Yang, Xueqi Ma, Wansheng Chen, Ying Xiao

**Affiliations:** ^1^ Research and Development Center of Chinese Medicine Resources and Biotechnology, The Ministry of Education (MOE) Key Laboratory for Standardization of Chinese Medicines, Institute of Chinese Materia Medica, Shanghai University of Traditional Chinese Medicine, Shanghai, China; ^2^ Shanghai Foreign Language School Affiliated to Shanghai International Studies University (SISU), Shanghai, China; ^3^ Jiangsu Kanion Pharmaceutical Co., Ltd., Lianyungang, China; ^4^ Department of Pharmacy, Changzheng Hospital, Naval Medical University (Second Military Medical University), Shanghai, China

**Keywords:** cofactor specificity, *Isatis indigotica*, lignan, secoisolariciresinol dehydrogenase, matairesinol, structural biology

## Abstract

Cofactors are crucial for the biosynthesis of natural compounds, and cofactor engineering is a useful strategy for enzyme optimization due to its potential to enhance enzyme efficiency. Secoisolariciresinol dehydrogenase (SIRD) was reported to convert secoisolariciresinol into matairesinol in an NAD^+^-dependent reaction. Here, a SIRD designated as *Ii*SIRD2 identified from *Isatis indigotica* was found to utilize NADP^+^ as the cofactor. To explore the structural basis for this unique cofactor preference, model-based structural analysis was carried out, and it was postulated that a variation at the GXGGXG glycine-rich motif of *Ii*SIRD2 alters its cofactor preference. This study paves way for future investigations on SIRD cofactor specificity and cofactor engineering to improve SIRD’s catalytic efficiency.

## Introduction

Cofactors are obligatory adducts in the catalytic machinery of numerous enzymes. As integral components of the holoenzymes, cofactors are imperatives for enzymatic and pathway functionality. Examples of cofactors include thiamine pyrophosphate (TPP) in pyruvate decarboxylase for yeast fermentation, flavin adenine dinucleotide (FAD) in acetyl-CoA-dehydrogenase for beta-oxidation of fatty acids, NADPH in adrenodoxin reductase for steroid hormone synthesis, etc. Without the effective participation of these cofactors, enzymes are incapable of efficiently transforming substrates into products. It is a challenging situation frequently encountered in metabolic engineering, which often involves introducing animal or plant metabolic pathways culled from nature into microorganisms with very different cellular environments and cofactor supplies (Akhtar and Jones, 2014). Besides, there is also the challenge of achieving cellular redox balance to enable biosynthesis at the maximum capacity, since cofactors can alter the intracellular redox state (Chen et al., 2018). To address these problems, researchers often resort to cofactor engineering. Previous successes in cofactor engineering include the optimization of vitamin C production. The formation of a vitamin C precursor, 2-keto-L-gluconic acid, is catalyzed by the NADPH-dependent 2,5-diketo-D-gluconic acid reductase (2,5-DKG). Banta et al. constructed 2,5-DKG mutants that could utilize both NADH and NADPH as the cofactor, eventually yielding an enzyme more active than the wild-type (Banta et al., 2002). This study highlights the significance of building cofactor specificity systems and identifying enzyme mutants with different cofactor preferences for cofactor engineering and pathway optimization.

**Figure 1:**
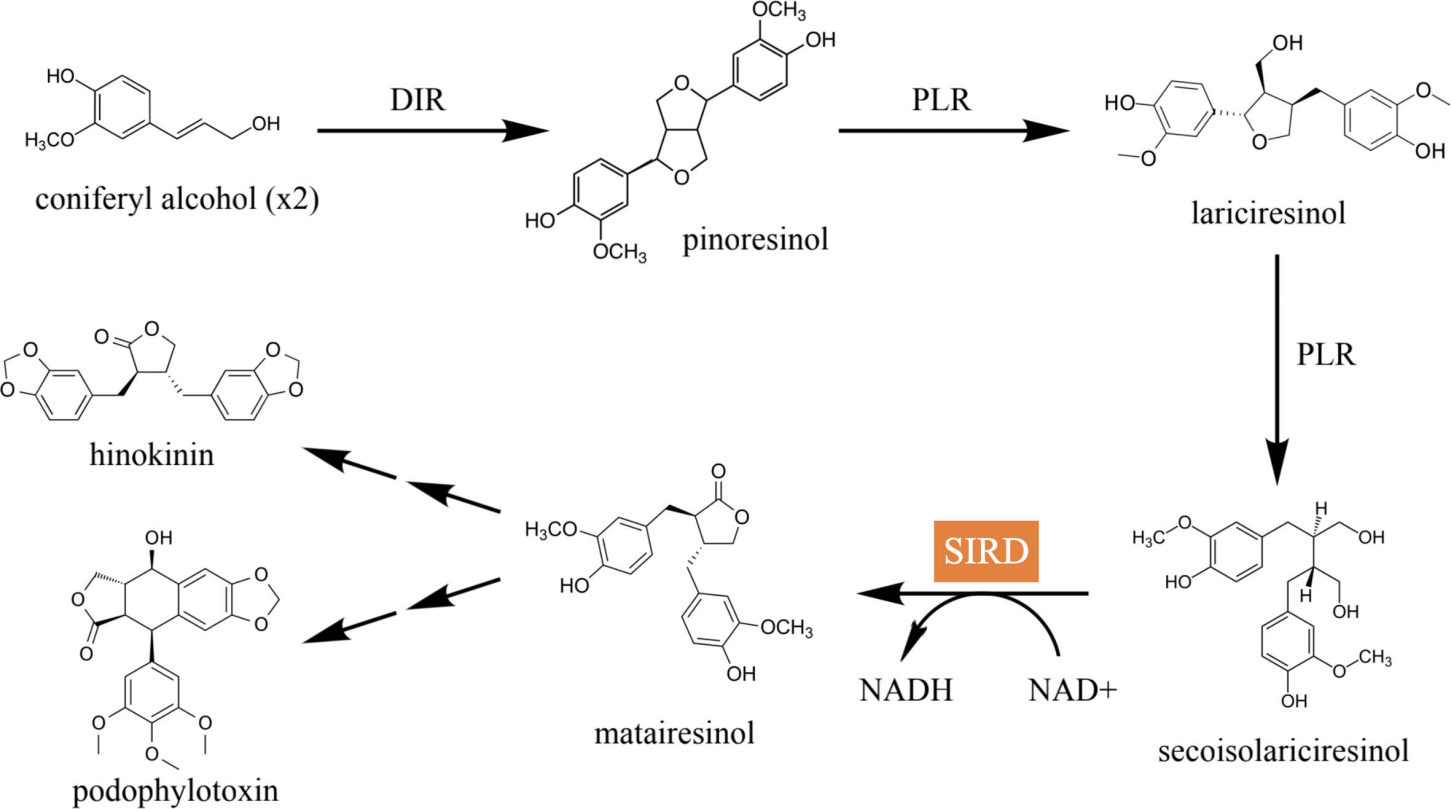
The major lignan biosynthetic pathway in plants. Coniferyl alcohol is dimerized by DIR into pinoresinol, which is then converted into lariciresinol and secoisolariciresinol in a step-wise manner catalyzed by PLR. Secoisolariciresinol is converted into matairesinol by SIRD, which is eventually converted into pharmacologically active compounds such as hinokinin and podophyllotoxin.

Secoisolariciresinol dehydrogenase (SIRD) is an NAD^+^-dependent enzyme that catalyzes the bioconversion of secoisolariciresinol into matairesinol. It is an important catalytic module for the biosynthetic pathways of lignans in plants. In one of the major biosynthetic pathways, coniferyl alcohol is dimerized by the plant dirigent protein (DIR) and converted into pinoresinol, which is then converted into secoisolariciresinol by pinoresinol-lariciresinol reductase (PLR) and matairesinol by SIRD in a stepwise manner (**Figure 1**) (Satake et al., 2015). The bioconversion catalyzed by SIRD lies in a key branch of the lignan biosynthetic pathways in plants; it determines the structural backbones of downstream bioactive lignans such as the anticancer podophyllotoxin and the anti-cancer, anti-inflammatory, and antimicrobial hinokinin, and hence it contributes to the structural and biological diversity of plant lignans (Marcotullio et al., 2014; Shah et al., 2021). Therefore, identifying efficient SIRD modules and expounding their mode of catalysis is essential for lignan biosynthetic engineering. In addition, matairesinol exhibits diverse biological activities such as anti-cancer, anti-oxidative, and immunoregulatory effects (Schröder et al., 1990), underscoring the importance of investigating SIRD’s catalysis for enhancing matairesinol production efficiency for future medical applications (Su et al., 2015; Wu et al., 2021).

Previous studies on SIRD focused on elucidating its catalytic functions. Shen et al. verified the catalytic functions of two *Dysosma versipellis* SIRDs (*Dv*SIRD) (Shen et al., 2016); Xia et al. and Arneaud et al. achieved the functional expression of *Podophyllum peltatum* (*Pp*SIRD) in *Escherichia coli* and *Pichia pastoris*, respectively (Xia et al., 2001; Arneaud and Porter, 2015), and the structure of *Pp*SIRD was determined with X-ray crystallography (Youn et al., 2005). Additionally, Decembrino et al. assembled three plant enzymes including *Pp*SIRD in *E. coli* and achieved the production of the podophyllotoxin precursor pluviatolide, highlighting SIRD’s future application in mass-producing the cancer drug precursor podophyllotoxin (Decembrino et al., 2020). All the previously reported SIRDs catalyze a strictly NAD^+^-dependent bioconversion: (1) The catalytic triad of Ser^153^, Lys^171^, and Tyr^167^ scaffolds NAD^+^ throughout the catalysis, and NAD^+^’s binding to Tyr^167^ favors Tyr^167^’s deprotonation. (2) Following substrate deprotonation and intramolecular hydride transfer, the intermediate lactol is formed and NAD^+^ is reduced into NADH. (3) NADH is released from a triad and another NAD^+^ binds the triad for the subsequent conversion of lactol into matairesinol in an analogous manner (Moinuddin et al., 2006).

*Isatis indigotica* Fort., belonging to the family *Cruciferae*, is a prevalent Chinese medicinal herb. Bioactive lignans and their corresponding derivatives have been identified as the major active ingredients of *I. indigotica.* In our previous study, notable progress has been made in understanding the biosynthetic pathway and regulatory mechanism of lignans in *I. indigotica* (Feng et al., 2021). Here, four SIRD genes were first identified in the *I. indigotica* genome. In particular, *Ii*SIRD2 was able to catalyze an NADP^+^-dependent conversion of secoisolariciresinol into matairesinol, which presents the first report of an NADP^+^-dependent SIRD. To fully understand the structural basis of its catalytic features, protein models were constructed using the *Pp*SIRD crystal structure as a template, revealing some unique features of *Ii*SIRD2. Based on molecular docking results, it was postulated that a variation at its GXGGXG motif enhances its affinity to NADP^+^ as the cofactor.

## Materials and methods

### Materials

The chemicals used in the experiments were reagent or High-Performance Liquid Chromatography (HPLC) grade. Restriction enzymes were purchased from New England BioLabs; RNA extraction kit was purchased from TransGen Biotech; cDNA synthesis kit and one-step cloning kit were purchased from Novo Protein Scientific (Shanghai); PCR kit was purchased from Toyobo Biotech; Premix TaqTM DNA polymerase was purchased from TaKaRa Bio; Taq master mix was purchased from Shanghai Wonton Biotech. *I. indigotica* was planted at Shanghai University of Traditional Chinese Medicine (SHUTCM), and two-month-old plants were used for target gene cloning.

### Identification of candidates genes

The whole genome of *I. indigotica* was used in this process (data unpublished). The TBtools program (https://github.com/CJ-Chen/TBtools) was used for sequence blasting. The “blast several sequences to a big database” function was used (outfmt: Table, NumofThreads: 2, E-value: 1e-5, NumofHits: 500, NumofAligns: 250). The protein sequence of five functional SIRDs were retrieved from GenBank, including *Ps*SIRD (GenBank ID: ALD51315.1), *Dv*SIRD (GenBank ID: ACB87357.1), *Dp*SIRD (GenBank ID: AHB18702.1), *Sh*SIRD (GenBank ID: ABN14311.1), and *Dt*SIRD (GenBank ID: ABD78859.1). Blasting these five SIRD protein sequences to the total protein database of *I. indigotica* using a tBLASTn algorithm, chromosome locations of all the hits were acquired. The “fasta extract” function in TBtools was then used to extract the protein sequences of these hits, and the acquired sequences were run against the SWISS-PROT protein database (as query sequences) in NCBI (https://www.ncbi.nlm.nih.gov/). The *I. indigotica* proteins matched with the reported functional SIRDs or the short-chain dehydrogenase family in the SWISS-PROT database were selected as SIRD candidates. Phylogenetic relationships were analyzed in MEGA 6.06 (https://www.megasoftware.net/) using the maximum likelihood method with the pairwise deletion option. Tree reliability was estimated using a bootstrap analysis of 1000 replicates.

### Cloning of the *I. indigotica* candidate gene

*I. indigotica* leave tissues were collected and frozen by liquid nitrogen and ground to fine powders. The total RNA of *I. indigotica* was then isolated using TRIzol reagent and then reverse-transcribed into cDNA according to the manufacturers’ instructions. Primers were designed as SIRD_CDS to amplify all the candidate genes in PCR using the *I. indigotica* total cDNA as the template (**Table S1**). The PCR products were separated in a 1% agarose gel. After cloning the amplified gene products into pMD™19-T, it was transformed into *E. coli* Top10 strain and sequenced by Sangon Biotech. Then, the candidate genes were amplified using primers SIRD_32a (**Table S1**) in PCR and cloned into the expression vector pET-32a at the *Not*I and *Xho*I restriction sites, thus generating 32a-*Ii*SIRD constructs. The constructs and the pET-32a control were transformed into *E. coli* expression strain BL21 for heterologous expression.

### Heterologous expression and protein purification

*E. coli* was grown overnight with shaking at 200 rpm in LB medium with 100 mg/L of ampicillin (LB Amp medium) and then inoculated into 500 ml of fresh LB Amp medium under the aforementioned condition. When the culture was grown to an optical density at 600 nm (OD600) of 0.6, 0.5 mM isopropyl β-D-1-thiogalactopyranoside (IPTG) was added to induce expression. The culture underwent 48 h of induction at 18°C with shaking at 80 rpm and bacteria were harvested by centrifugation at 7830 rpm for 5 min at 4°C. Cell pellets were resuspended in 10 ml of suspension buffer (50 mmol/L Tris-HCl, 20% glycerol, 10 mmol/L 2-mercaptoethanol, pH 8.0) for every 200 ml of culture. To break the cell, an ultrasonic cell crusher was employed under the following condition: power 35%, ultrasound 3 s, gap 2 s, 50 Hz, 10 min, maximum temperature 10°C. The suspensions were next centrifuged at 7830 rpm for 15 min at 4°C and impurities were removed using a 0.22 μm membrane filter to yield crude protein extracts. Protein purification was performed using a His Spin Trap column following the manufacturer’s instructions (GE Healthcare). The purity of the His-tag-fused *Ii*SIRDs was examined by 12% SDS–PAGE, and the protein concentration was determined by the Bradford method (Bradford, 1976) with bovine serum albumin (BSA) as the standard.

### Enzyme assay and LC-MS analysis

The assay mixture (100 μL) consisting of Tris buffer (20 mM, pH 8.8), 1 mM NADP (sodium salt), 500 μm secoisolariciresinol, and 10 μg of purified protein was incubated at 30°C with shaking at 300 rpm for 12 hours (overnight), and the pET-32a vector was used as negative control. The reaction mixtures were analyzed by LC-MS using a triple-quadrupole mass spectrometer (Model 6410, Agilent, Santa Clara, CA) according to the method previously reported (Xiao et al., 2015).

### Structural modeling and molecular docking

First, the primary structure of *Ii*SIRD was analyzed based on its alignment with five other functional SIRDs and five short-chain dehydrogenases/reductases in the Jalview program (https://www.jalview.org/): *Ps*SIRD (GenBank ID: ALD51315.1), *Dv*SIRD (GenBank ID: ACB87357.1), *Dp*SIRD (GenBank ID: AHB18702.1), *Sh*SIRD (GenBank ID: ABN14311.1), *Dt*SIRD (GenBank ID: ABD78859.1), the Rv2002 gene product of *Mycobacterium tuberculosis* (PDB ID: 1NFF), 3 α, 20 β-hydroxysteroid dehydrogenase from *Streptomyces exfoliatus* (*Se*HSD; PDB ID: 2HSD), R-alcohol dehydrogenase from *Lactobacillus brevis* (*Lb*RADH; PDB ID: 1NXQ), glucose dehydrogenase from *Priestia megaterium* (*Pm*GluDH; PDB ID: 1GCO), and 3-hydroxyacyl-CoA dehydrogenase from *Rattus norvegicus* (*Rn*HAD; PDB ID: 1E6W). Then, the secondary structure of *Ii*SIRD was predicted on the PSIPRED workbench web-server (http://bioinf.cs.ucl.ac.uk/psipred/). Furthermore, protein models were constructed using the SWISS-MODEL web-server (https://swissmodel.expasy.org/) based on the previously reported crystal structures of *Podophyllum* SIRD (*Pp*SIRD) in the apoenzyme form (PDB ID: 2BGK), NAD^+^-bound holoenzyme form (PDB ID: 2BGL), and the NAD^+^-matairesinol-bound form (PDB ID: 2BGM) (Arneaud and Porter, 2015). It is important to note that the sequence identity between *Pp*SIRD and the modeled enzyme is calculated to be 45.91% on the SWISS-MODEL web-server, and considering that a protein sequence with more than 30% similarity to a known structure may frequently be predicted with the accuracy of a low-resolution X-ray structure (Xiang, 2006), the structural prediction is likely to be reliable. Then, protein tertiary structures were visualized in the PyMol program (https://pymol.org/2/). To examine the cofactor preference of *Ii*SIRD, molecular docking was performed using AutoDock Vina (https://vina.scripps.edu/). Moreover, the root-mean-square deviation of atomic positions (RMSD) was calculated using the SuperPose web server (http://superpose.wishartlab.com/) to determine the effect of point mutations on the enzymatic structure. Finally, to analyze the glycine-rich motif and the enzyme pocket, pocket-cavity search and volume prediction were carried out using the POCASA 1.1 web-server (http://g6altair.sci.hokudai.ac.jp/g6/service/pocasa/), and loop refinement was carried out using Modeller (https://salilab.org/modeller/).

## Results and discussion

### Identification of SIRD gene candidates

Four SIRD gene candidates were identified from the genome annotation pool of *I. indigotica*, and they were translated into amino acid sequences (**Tables S2**, **3**). The upstream and downstream sequences of the four SIRD gene candidates were presented in **Table S4**. A phylogenic tree was then constructed together with five functional SIRD proteins previously reported (**Figure S1**). It was noticed that the *I. indigotica* candidates (*Ii*SIRDs) and the functional SIRDs cluster independently on the phylogenic tree, hinting that *I. indigotica* candidates might not share the same catalytic behavior with the known SIRDs.

### Characterization of *Ii*SIRD activity

**Figure 2:**
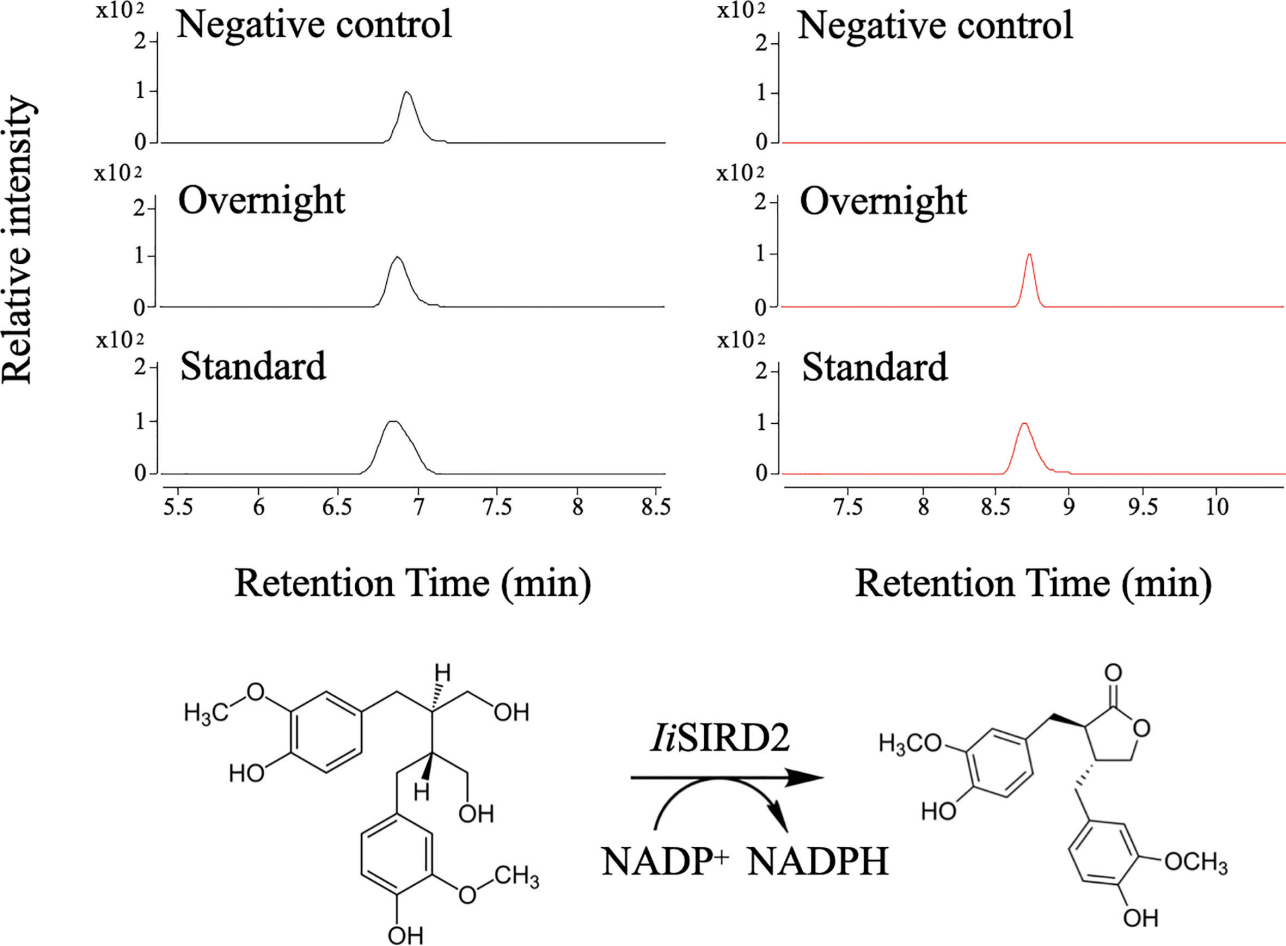
Biochemical assays for *Ii*SIRD2 function. Matairesinol was detected in the enzyme assay mixture, proving that *Ii*SIRD2 catalyzes an NADP^+^-dependent reaction of converting secoisolariciresinol into matairesinol.

SDS-PAGE analysis of *Ii*SIRD1, *Ii*SIRD2, *Ii*SIRD3, and *Ii*SIRD4 expressions in *E.coli* revealed that the recombinant SIRDs have a molecular mass of 40~50 kDa (Fig. S2), which is close to the predicted molecular mass. LC-MS analysis results of enzyme assays showed that secoisolariciresinol was successfully converted into matairesinol by *Ii*SIRD2 using NADP^+^ as the cofactor (**Figure 2**). The other three candidates show no enzymatic activity. Furthermore, *IiSIRD2’s* gene sequenc*e* has been uploaded to GenBank (GenBank ID: OM777730).

### The mechanism underlying the cofactor specificity of *Ii*SIRD2

To understand why *Ii*SIRD2 is capable of catalyzing an NADP^+^-dependent reaction different from other functional SIRDs, protein models were constructed and structural analyses were carried out as follows.

**Figure 3:**
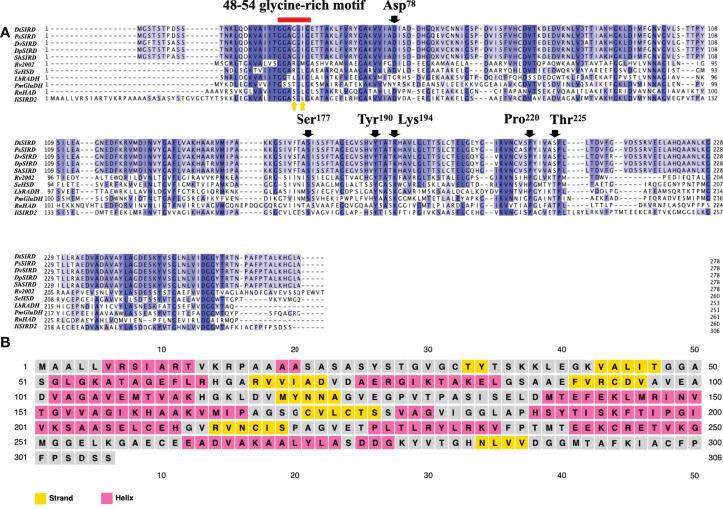
Primary and secondary structure of *Ii*SIRD2. **(A)** *Ii*SIRD2 is conserved at Ser^177^, Tyr^190^, Lys^194^, Asp^78^, Pro^220^, and Val^225^. However, *Ii*SIRD2 displays a glycine-rich motif (GXSGLG) different from the five functional SIRDs (GXGGIG). **(B)** Secondary structure prediction of *Ii*SIRD2.

*Primary and Secondary Structure Analysis*: Protein alignment (**Figure 3A**) revealed that similar to the five functional SIRDs, *Ii*SIRD2 is conserved at the highly conserved catalytic triad consisting of Ser^177^, Tyr^190^, and Lys^194^, which was suggested to be essential for catalysis to occur (Youn et al., 2005). *Ii*SIRD2 is also conserved at Asp^78^ which accounts for SIRDs’ specificity for NADH instead of NADPH (Youn et al., 2005). Pro^220^ and Val^225^, two sites involved in cofactor stabilization are also conserved for *Ii*SIRD2 (Youn et al., 2005). Another important motif conserved for NAD^+^-binding enzymes is the glycine-rich motif (GXGGXG; amino acid position 48~54), which is known to bind the pyrophosphate group of NAD^+^ (Youn et al., 2005). Surprisingly, *Ii*SIRD2 exhibits a deviation (GXSGXG) from the conventional pattern (GXGGXG) at this motif (**Figure 3A**, **Table 1**), substituting serine for the glycine at site 51. Additionally, the five reported functional SIRDs contain isoleucine at the glycine-rich motif (GXGGIG), while *Ii*SIRD2 replaces isoleucine with leucine at site 53 (GXSGLG). Although it was previously reported that SIRD has specificity toward NAD^+^ but not NADP^+^, it was noticed from the enzyme assay result that *Ii*SIRD2 can utilize NADP^+^ as its cofactor to catalyze the reaction, leading to the question of whether its unique glycine-rich motif accounts for the special cofactor preference to *Ii*SIRD2. Then, the secondary structure of *Ii*SIRD2 was predicted (**Figure 3B**): similar to the reported *Podophyllum peltatum* SIRD (*Pp*SIRD) (Arneaud and Porter, 2015), *Ii*SIRD2 monomer adopts an α/β domain structure, containing 7 β-strands flanked by 8 α-strands, reminiscent of the Rossmann fold typical for NAD(P)(H) binding.

**Table 1:** Variations of the glycine-rich motif (Amino acid variations are marked in red).

Genes	The Glycine-rich Motif (48-54)
*DtSIRD*	GGAGGIG
*PsSIRD*	GGAGGIG
*DvSIRD*	GGAGGIG
*DpSIRD*	GGAGGIG
*ShSIRD*	GGAGGIG
*IiSIRD2*	GGASGLG

*Structural Modeling and Molecular Docking Studies*: S51G and L53I were introduced to *Ii*SIRD2 to create models of three protein mutants: *Ii*SIRD2-S51G, *Ii*SIRD2-L53I, and *Ii*SIRD2-S51G/L53I. Their binding affinity to NAD(P)^+^ was tested to investigate the effect of these two sites on NAD(P)^+^ binding affinities. To guarantee the reliability of the data, molecular docking was performed in triplicate and the average values were computed. The molecular docking results are shown in **Table 2**. In general, *Ii*SIRD2 (WT) exhibits a stronger affinity to both NADP^+^ and NAD^+^ compared with *Pp*SIRD. The reason for this alteration in cofactor preference perhaps lies in its variation at sites 51 and 53: introducing S51G to *Ii*SIRD2 decreases its affinity to NADP^+^ significantly and slightly lowers its affinity to NAD^+^, suggesting that Ser^51^ enhances *Ii*SIRD2’s affinity to both coenzymes, especially NADP^+^, thus explaining the enzyme assay result. Additionally, it is interesting that L53I only slightly alters binding affinity to both cofactors, while both S51G and S51G/L53I lower NAD^+^ binding affinity by ~0.3 kcal/mol, indicating that Leu^53^ might play a minor role in determining cofactor preference compared with Ser^51^. Based on molecular docking results, it is safe to postulate that site 51 within the glycine-rich motif is essential in determining NAD^+^-binding affinity, while site 53 is comparatively less important. Furthermore, the RMSD value upon point mutation was determined using the SuperPose web server to evaluate the magnitude of structural change caused by the introduction of S51G and L53I to *Ii*SIRD2. In line with molecular docking results, S51G has a high RMSD value of 3.33, whereas L53I has an insignificant RMSD value of 0.84, showing that site 51 is more relevant in defining the structure of the enzyme. Replacing glycine with serine at site 51 might serve to enhance the enzyme’s affinity to NADP^+^ while substituting isoleucine for leucine at site 53 might counterbalance that change by slightly lowering its affinity to NADP^+^. In brief, the unique glycine-rich motif of *Ii*SIRD2 offers a new perspective on how such a motif correlates with SIRD’s cofactor preference and explains the NADP^+^ specificity of *Ii*SIRD2.

**Table 2:** Molecular docking results (Average values calculated from triplicate results were highlighted in bold).

		1	2	3	average
*Pp*SIRD (2BGL)	NAD+	-9	-8	-8.6	**-8.53**
	NADP+	-8.1	-8.5	-8.1	**-8.23**
*Ii*SIRD (WT)	NAD+	-10.1	-9.6	-9.1	**-9.60**
	NADP+	-9.8	-10.2	-9.5	**-9.83**
S51G	NAD+	-9.2	-9.3	-9.5	**-9.33**
	NADP+	-8.7	-9.3	-8.7	**-8.90**
L53I	NAD+	-9.6	-9.7	-9.6	**-9.63**
	NADP+	-9.8	-9.4	-10.1	**-9.77**
S51G, L53I	NAD+	-9.4	-9.1	-9.4	**-9.30**
	NADP+	-8.7	-8.7	-8.7	**-8.70**

**Figure 4:**
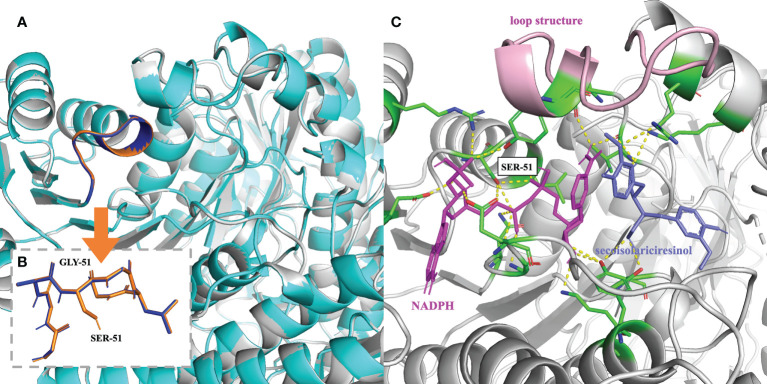
Mechanism underlying the cofactor specificity of *Ii*SIRD2. **(A)** Superimposition of *Ii*SIRD2 (gray) and *Ii*SIRD-S51G (cyan). The glycine-rich motif is highlighted (*Ii*SIRD-S51G: blue; *Ii*SIRD: orange). **(B)** Superimposition of the glycine-rich motif of *Ii*SIRD-S51G (blue) and *Ii*SIRD2 (orange). Steric hindrance caused by Ser^51^ may alter the way NAD(P)^+^ binds the motif. **(C)** Structure of *Ii*SIRD (gray) bound with NADPH (magenta) and secoisolariciresinol (slate). Yellow-dotted lines represent potential hydrogen bonds, and key residues including Ser^51^ are marked in green. The loop structure at the pocket entry is marked in pink.

*Analysis of the Glycine-rich Motif and the Enzyme Pocket*: Based on the above analysis, attention was then dedicated to the glycine-rich motif (**Figures 4A, B**) and the substrate-binding pocket (**Figure 4C**). At the glycine-rich motif, Ser^51^ pokes out from the traditional structure, leading to a conformational change that might alter the way NAD(P)^+^ binds the motif due to steric hindrance (**Figures 4A, B**). NAD(P)^+^ might be able to bind the motif in a more favorable position, rendering NAD(P)^+^ the cofactor of *Ii*SIRD2. In brief, this conformational change offers some clues to the unique cofactor affinity of *Ii*SIRD2. The volume of the catalytic pocket was predicted. *Pp*SIRD (PDB ID: 2BGM) and *Ii*SIRD2 were predicted to have pocket volumes of 1340 and 1012, respectively (unit gird size of 1 Å). Interestingly, a closer look at the enzyme’s tertiary structures reveals a short loop structure at *Ii*SIRD2’s pocket gate (**Figure 4C**), and loop refinement with Modeller indicates that this structure may have a significant influence on the pocket structure, which warrants more examination in the future.

## Conclusions and perspectives

This study presents the first report of an NADP^+^-dependent SIRD. Protein model analysis and molecular docking studies revealed the role the glycine-rich motif plays in determining SIRD’s cofactor preference, shedding some light on SIRD’s catalytic mechanism. Since SIRD is an important entry point for downstream lignan synthesis, comprehending its catalytic behaviors is crucial for understanding the biological diversity of these health-protecting lignans.

The identification of the NADP^+^-dependent *Ii*SIRD2 and its unique glycine-rich motif pave way for future research on cofactor engineering, a nascent strategy of interest that might be employed to evolve other SIRDs to alter cofactor specificity for enzyme optimization. Besides the success of enhancing the efficiency of vitamin C production by altering the cofactor preference of the relevant enzyme (Banta et al., 2002), phosphite dehydrogenase mutants with an enhanced preference for NADP^+^ were recently demonstrated to be applicable to the establishment of an NADPH regeneration system for an NADPH-dependent reaction (Abdel-Hady et al., 2021), hinting that the SIRD cofactor specificity system enriched by the NADP^+^-dependent *Ii*SIRD2 might provide a promising alternative cofactor regeneration machinery for practical applications to reduce production costs and increase efficiency for many NADPH-dependent reactions. Finally, this study also contributes to the full elucidation of the *I. indigotica* lignan biosynthetic pathway and thus helps to prompt the possibility of a potentially efficient enzyme-based metabolic engineering for the large-scale production of health-protecting lignans including matairesinol and its derivatives.

To conclude, this study reports the first NADP^+^-dependent SIRD, providing theoretical support for fully expounding *Ii*SIRD2’s catalytic characteristics and molecular mechanism, paving way for cofactor engineering, and hence having important implications regarding the efficient bioconversion of matairesinol.

## Data availability statement

The original contributions presented in this study are included in the article/**Supplementary Material**. Further inquiries can be directed to the corresponding authors.

## Author contributions

XS, JG, YY, and XM carried out the experiments and analyzed data. XS and JF performed protein modeling and molecular docking studies. XS wrote the manuscript. XS, YX, and JG made manuscript revisions. YX and WC supervised the study. All authors contributed to the article and approved the submitted version.

## Funding

This work was sponsored by the National Natural Science Foundation of China (81874335, 32170402, and 31872665) and Shanghai local Science and Technology Development Fund Program guided by the Central Government (YDZX20203100002948).

## Conflict of interest

Author YY was employed by Jiangsu Kanion Pharmaceutical Co., Ltd.

The remaining authors declare that the research was conducted in the absence of any commercial or financial relationships that could be construed as a potential conflict of interest.

## Publisher’s note

All claims expressed in this article are solely those of the authors and do not necessarily represent those of their affiliated organizations, or those of the publisher, the editors and the reviewers. Any product that may be evaluated in this article, or claim that may be made by its manufacturer, is not guaranteed or endorsed by the publisher.

## Abbreviations

SIRD, secoisolariciresinol dehydrogenase; TPP, thiamine pyrophosphate; FAD, flavin adenine dinucleotide; 2,5-DKG, 2,5-diketo-D-gluconic acid reductase; DIR, dirigent protein; PLR, pinoresinol-lariciresinol reductase; *Ii*, *Isatis indigotica*; *Pp*, Podophyllum peltatum.
