# Supplementary material for: Identification and investigation of a novel NADP+-dependent secoisolariciresinol dehydrogenase from Isatis indigotica
Source: Front Plant Sci. 2022 Nov 2;13:1035121. doi: 10.3389/fpls.2022.1035121 (PMC9666873; doi:10.3389/fpls.2022.1035121)
Supplement: Supplementary file 1 [file DataSheet_1.pdf]

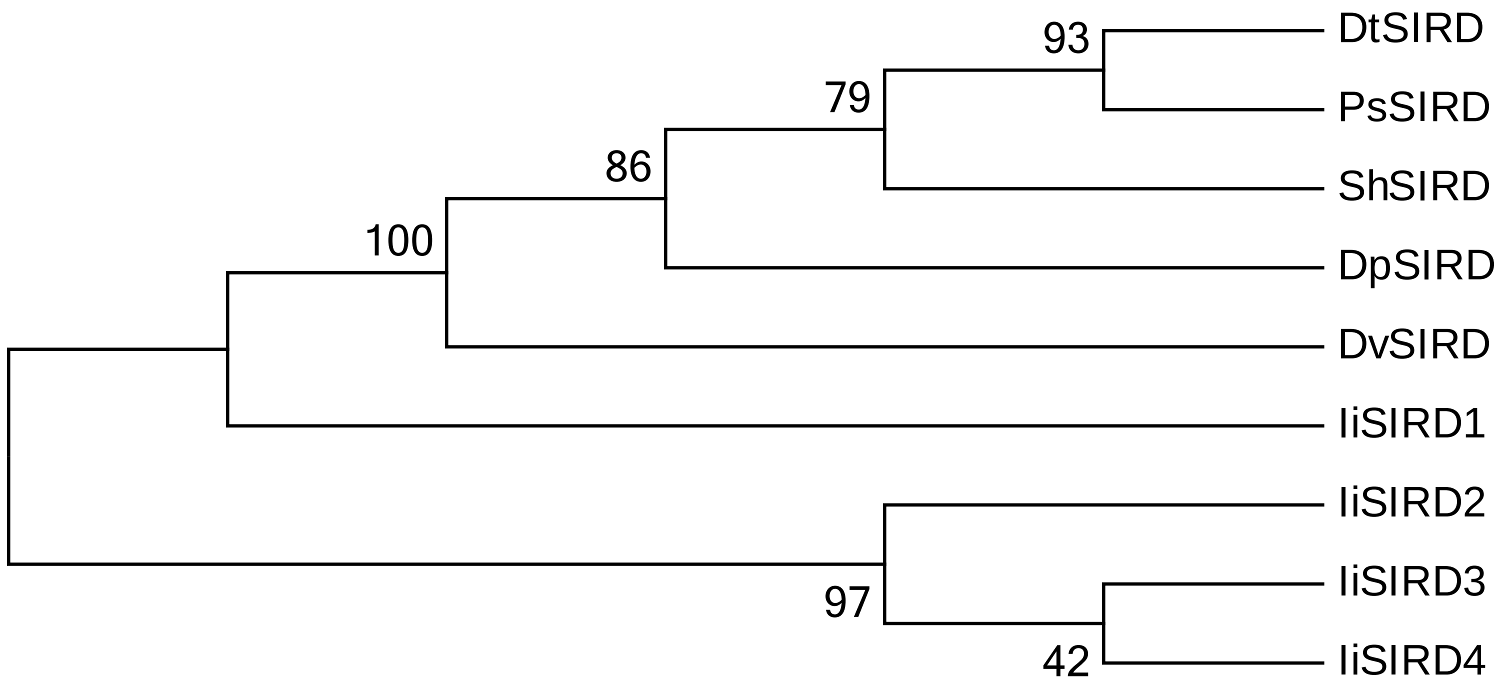

Figure S1. Phylogenic tree of the *I. indigotica* SIRD candidates with five functional SIRDs. The *Isatis indigotica* SIRD candidates cluster on the tree, suggesting possible functional differentiation from other SIRD.

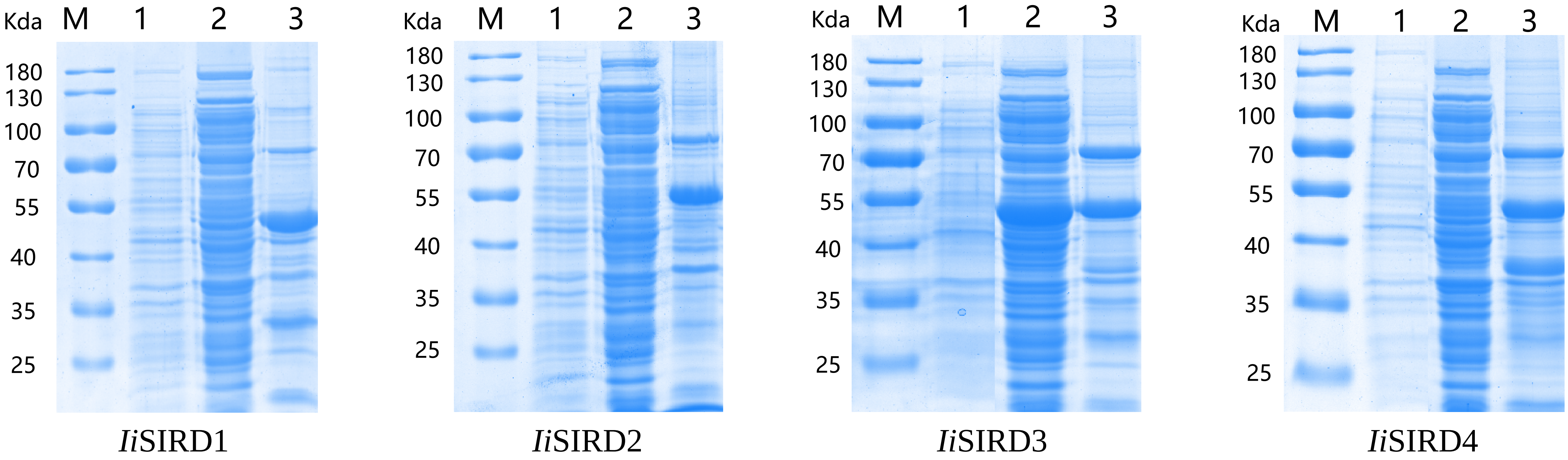

Figure S2. SDS-PAGE analysis of expression and purification of recombinant *Ii*SIRD1, *Ii*SIRD2, *Ii*SIRD3, and *Ii*SIRD4. The recombinant *Ii*SIRDs have a size of 40~55 kDa. Lanes: M, protein marker; 1, uninduced cells; 2, isopropyl β-D-thiogalactoside–induced cells; 3, purified recombinant protein.
